# Avian Haemosporidian Parasites in Three Wild Columbids from Germany

**DOI:** 10.3390/microorganisms13061305

**Published:** 2025-06-04

**Authors:** Yvonne R. Schumm, Celine Frank, Uta Gerz, Hannes Ruß, Benjamin Metzger, Petra Quillfeldt

**Affiliations:** 1 Department of Animal Ecology & Systematics, Justus Liebig University, Heinrich-Buff-Ring 26-32, 35392 Giessen, Germany; celine.frank@bio.uni-giessen.de (C.F.); uta.gerz@bio.uni-giessen.de (U.G.); hannes.russ@bio.uni-giessen.de (H.R.); 2 Independent Researcher, 26/1 Immaculate Conception Street, GZR 1141 Gzira, Malta; ben.lanius@gmail.com

**Keywords:** avian host, blood parasite infection, H/L ratio, haemosporidian lineage, European Turtle Dove

## Abstract

Birds are hosts to a diverse assemblage of vector-transmitted haemosporidian parasites. However, the true genetic diversity and many host–parasite interactions are still unknown, particularly in under-represented species groups such as wild doves and pigeons (Columbiformes). In this study, we examined the prevalence and lineage diversity of haemosporidian genera *Plasmodium*, *Leucocytozoon*, and *Haemoproteus* in three species of wild columbids, sampled in Germany. Examinations were performed by applying molecular methods (nested PCR and one-step multiplex PCR) and blood smear examination, and their respective advantages and disadvantages are discussed. In the case of the European Turtle Dove *Streptopelia turtur*, samples were collected along a west–east gradient throughout Germany, covering migratory birds from the Western and Central-Eastern flyway of this species. Although no infection was detected in the Stock Dove *Columba oenas* samples, 53% of Turtle Dove and 86% of Common Woodpigeon *Columba palumbus* harbored a parasite of at least one haemosporidian genus, revealing previously unknown lineage–host interactions. We were not able to demonstrate a correlation between infection status (presence or absence of infection based on PCR results) and parasitemia with condition based on the heterophil to lymphocyte ratio (H/L ratio). Neither lineage occurrence nor prevalence of infection followed any geographically specific patterns. Thus, haemosporidian lineages found in Turtle Doves could not be used as a marker of geographic origin that would allow the tracking of their nonbreeding distribution.

## 1. Introduction

Emerging infectious diseases are increasingly cited as a relevant threat to wild and domestic animals as well as humans, with parasites being a common and integral component of ecosystems [1,2,3,4]. Parasites are ubiquitous and occur in all food webs at all trophic levels, potentially impacting their host significantly in numerous ways, such as behaviorally, physiologically, morphologically, or reproductively [2,5]. However, the impact of parasitism extends beyond the costs imposed on their individual host, as mounting evidence suggests that parasites play an important role in structuring ecological communities [2]. While impacts on an individual level are often well documented (e.g., [3,6,7]), consequences on greater scales, like population levels or across zoogeographical realms, are rather poorly understood [4,8]. The assessment of the impacts of diseases on wild animal populations is challenging, as often little is known about a given species’ parasite composition, because the detection and diagnosis of pathogens and diseases in wildlife are still facing constraints [4,9].

Thereby, infectious diseases may not only be a threat to endangered or endemic animal species (e.g., [10,11]), but also to species with larger population sizes [12]. For instance, infections with *Plasmodium relictum*, the most common agent of avian malaria [13], are suspected to influence population dynamics of House Sparrow *Passer domesticus* in suburban areas, which may be connected to an observed general decline of this species [14]. Generally, the avian haemosporidian parasites (Apicomplexa: Haemosporida) of the genus *Plasmodium*, known as avian malaria, and related pathogens *Leucocytozoon* and *Haemoproteus* have been associated with affecting host fitness and survival negatively in susceptible bird species [15,16]. However, because parasitemia varies during infection (acute vs. chronic stage), and the character and severity of symptoms depend on various factors, such as the specific parasite lineage or host immunity, it is difficult to assess the fitness effects of haemosporidian infections in nature [17,18].

Changes in the leucocyte profile can be used as an approximation for the immune response to parasitic infection [17]. Examining the heterophil-to-lymphocyte ratio (H/L ratio) might be a suitable proxy for parasitemia, as individuals that are exposed to various stressors often increase their heterophil production and redistribute lymphocytes out of the blood, thus increasing their H/L ratio [19]. Generally, the H/L ratio is expected to increase in response to infection [20,21]. However, other studies suggest the opposite, i.e., a reduction in the H/L ratio associated with haemosporidian infection [22,23], or found no significant change in the ratio [24,25].

While the haemosporidian parasite genera differ in their biology, morphology, and ecology, all depend on a bird host and a hematophagous dipteran vector to complete their life cycle. The unicellular blood parasites, which are widespread and infect a great variety of hosts, have been the focus of an increasing number of studies, primarily in naturally infected wild birds [16,26], however, with a certain bias toward passerine birds, while research on non-passerine host species is underrepresented [27,28,29]. Focused sampling of non-passerine host species, though, is crucial to identify new taxa and to improve our understanding of haemosporidian phylogeny [27,30].

Besides studies on Feral Pigeons, only a few recent publications investigate haemosporidian parasites in free-ranging doves and pigeons (Columbiformes) (e.g., [31,32,33,34]). Columbiformes form one of the oldest and most diverse extant lineages of birds. Despite their relatively conserved anatomy and morphology, species of Columbiformes display a wide range of variations in their ecological adaptations [35,36]. Aside from Rock and domesticated Feral Pigeon (*Columba livia* and *C*. *livia f*. *domestica*, respectively) and endemic species on the Canary Islands, only four species of native wild Columbiformes breed in Europe, namely, the Common Woodpigeon *Columba palumbus*, the Eurasian Collared Dove *Streptopelia decaocto*, the European Turtle Dove *Streptopelia turtur*, and the Stock Dove *Columba oenas*. In a previous study, we screened European Turtle Doves from several European countries as well as Common Woodpigeons and Stock Doves from Germany for haemosporidian parasites, revealing an overall prevalence (the proportion of infected individuals in a host population) of 42% [33]. Stock Doves showed the lowest overall prevalence (4%) compared to higher prevalence in Turtle Doves (49%) and Woodpigeons (62%), potentially because, as cavity-nesters, Stock Doves might be less exposed to dipteran vectors [31]. Woodpigeons (residents or short-distance migrants) had a higher infection prevalence with *Leucocytozoon* and a lower prevalence of *Plasmodium* and *Haemoproteus*, compared to long-distance migrating Turtle Doves, which showed a higher prevalence of haemoproteid parasites. This pattern likely occurs because for birds breeding in the northern hemisphere, *Leucocytozoon* parasites are often transmitted at the breeding grounds, whereas for long-distance migrants, haemoproteid parasite transmission primarily occurs in the wintering areas and along the migration routes [26,37].

Previous works on Turtle Doves breeding in Europe have suggested a migratory divide for the species within the European breeding ground, which is reflected in their different migratory flyways [38,39]. Accordingly, mirroring the Western and Central-Eastern flyway, different wintering areas for Turtle Doves are assumed for the Western, Central, and Eastern Sub-Saharan regions. Schumm et al. [33] found no significant differences in haemosporidian prevalence between the flyways in Turtle Dove samples, originating from seven European countries. However, the sample size for Turtle Doves breeding in Germany, a country with individuals from both the Western and Central-Eastern flyway, was rather low (*n* = 13).

In the present work, we focus solely on Turtle Doves that spend the breeding period within Germany, with samples collected in particular along a west–east gradient across different German federal states. Applying molecular and microscopic analysis methods, we aim to test the following hypotheses:

(I). Congruent with the pattern in samples from different European countries, Turtle Doves and Woodpigeons, sampled in Germany, possess a similar haemosporidian parasite prevalence, whereas Stock Doves have a lower infection prevalence.
(II). Turtle Doves along a west–east gradient within Germany, across the presumed migratory divide, have a similar prevalence but different haemoproteid parasite lineages, reflecting differences in nonbreeding regions.
(III). The H/L ratio of individuals with and without haemosporidian parasite infection does not vary significantly if only chronic infections, characterized by a low parasitemia (i.e., proportion of erythrocytes infected with haemosporidian parasites), are diagnosed.

## 2. Materials and Methods

### 2.1. Sample Collection and Preparation

A small amount of blood was sampled from three species of Columbiformes by venipuncture of the brachial vein (Common Woodpigeon *Columba palumbus n* = 7, Stock Dove *Columba oenas n* = 13, and European Turtle Dove *Streptopelia turtur n* = 47). All sampled individuals were caught within the breeding season of the years 2023 (2 June to 5 July) and 2024 (30 May to 17 July) within Germany in different federal states along a west-to-east transect Hesse, Thuringia, Saxony-Anhalt, and Brandenburg (Table 1, Figure S1). Except for one Turtle Dove and one Stock Dove, which hatched in the calendar year of capture, all other sampled individuals hatched in at least the previous year (determined by plumage characteristics).

One part of the blood was stored on Whatman FTA cards (Whatman^®^, Buckinghamshire, UK), and the remaining part was used to create whole-blood smears on object slides. Subsequently, the smears were fixed in 100% methanol for 30 s and then stained with a Giemsa solution prepared with buffer pH 7.0 (ratio 1:5) for 30 min. If the staining was insufficiently intense, the staining process was repeated for an additional 30 min.

For DNA isolation, a 3 × 3 mm piece of an individual sample was cut out of the FTA card, and the DNA was extracted according to the ammonium-acetate protocol by Martínez et al. [40] and purified with NZYGelpure columns (NZYTech, Lisboa, Portugal). Concentration and purity of the isolated DNA were quantified by using a NanoDrop2000c UV–Vis spectrophotometer (NanoDrop Technologies, Thermo Scientific^TM^, Waltham, MA, USA). Samples with concentrations exceeding 120 ng/µL were diluted to 20 ng/µL. The sex of each individual was determined by PCR analysis according to Griffiths et al. [41].

### 2.2. Parasite Detection via PCR Method

We used two types of PCR assays to determine haemosporidian parasite presence in the host blood samples and to disentangle co-infections. DNA from birds with known haemosporidian infection (for *Haemoproteus*-*Plasmodium*-specific PCR, a Woodpigeon sample infected with AFR119, and for *Leucocytozoon*-specific PCR, a Turtle Dove sample infected with COLIV04) and deionized water were included in PCR runs as positive and negative controls, respectively.

The first approach was through a nested polymerase chain reaction (PCR) that targets an approx. 480 base pair (bp) region of the cytochrome *b* gene (cyt *b*; [42]; Table S1). The second approach was a PCR assay (one-step multiplex PCR) for simultaneous detection of *Plasmodium*, *Haemoproteus*, and *Leucocytozoon* following Ciloglu et al. [43] using equimolar concentrations of three primer sets PMF/PMR, HMF/HMR, and LMF/LMR in a single-reaction tube (Table S1). These target different-sized fragments, namely, approx. 380 bp fragment of the non-coding region of *Plasmodium* mtDNA, approx. 530 bp fragments between the 5′ end of cyt *b* and a non-coding region of mtDNA of *Haemoproteus*, and approx. 220 bp fragments of the cytochrome c oxidase subunit 1 (COX1) gene of *Leucocytozoon* (Table S1). Both PCR assays (see [33] for detailed PCR setup and cycling conditions) were performed on Biometra TOne Cyclers (Analytik Jena GmbH+Co. KG, Jena, Germany).

PCR amplicons were visualized using QIAxcel Advanced (Qiagen, Hilden, Germany) high-resolution capillary gel electrophoresis, and samples of the nested PCR assay rendering a clear peak (*n* = 31 host individuals, in detail: *n* = 15 PCR amplicons for *Leucocytozoon* and *n* = 26 for *Haemoproteus*/*Plasmodium*) were sent to be Sanger sequenced bidirectional by Microsynth-Seqlab (Microsynth AG, Balgach, Switzerland).

### 2.3. Phylogenetic and Statistical Analyses

The Sanger-sequenced forward and reverse sequences were assembled and assessed for co-infections [44] in CLC Main Workbench v. 23.0.2 (CLC Bio, Aarhus, Denmark). Of the 15 samples positive for *Leucocytozoon* and 26 samples positive for *Haemoproteus*/*Plasmodium* sent in for sequencing, 14 and 19 samples, respectively, could be clearly assigned to one distinct haemosporidian parasite lineage. The one *Leucocytozoon*-positive sample, which returned no result when Sanger sequenced, i.e., only unknown nucleotides, was negative when the PCR was repeated and was thus considered negative for further evaluation. In four cases for *Haemoproteus*/*Plasmodium*-positive samples, Sanger sequencing resulted in too short fragments for lineage determination, but blast search in MalAvi and/or NCBI GenBank resulted in parasite genus determination: three samples belonged to *Haemoproteus* spp. infections and one sample to a *Plasmodium* spp. infection. A PCR repetition of the remaining three samples (one Turtle Dove and two Woodpigeon samples) produced no bands, i.e., negative samples; thus, these individuals were classified as free of *Haemoproteus*/*Plasmodium* infections (Table 1).

Resulting consensus sequences were aligned to reference sequences stored in the MalAvi database [45] using the built-in BLASTN 2.5.9 tool in order to identify parasite genus and lineage. Individual lineages differ by one or more nucleotides in the cyt *b* fragment [42,45]. BioEdit v. 7.7.1 [46] was used to align 478 bp long cyt b fragments (*n* = 34) for network construction. Construction of a lineage network by the medium joining network method was realized with PopART v1.7 [47]. A second network was constructed with the consequences from this work (*n* = 34) and from a previous study on columbiform birds (*n* = 129, [33], GenBank accession numbers MT888848-60). A sequence found in a hitherto unknown host species and a new lineage were deposited in GenBank under accession numbers PV402400 and PV402401, respectively.

All statistical analyses were performed with R Statistical Software v4.4.1 [48]. Infection status as determined by nested PCR expressed as binomial contrast: presence or absence of infection, i.e., a positive or negative tested sample, was used for statistical tests unless stated otherwise. The Shapiro–Wilk test was applied to check the data for normal distribution to decide the respective subsequent statistical tests.

### 2.4. Parasite Evidence via Examination of Blood Smears

To check for the presence of extra- and intracellular parasite development stages, stained blood smears (*n* = 66, Table 1) were examined at a 1000× magnification (oil immersion) with a light microscope (Model B4, Exacta & Optech GmbH, Munich, Germany). The intensity of parasitemia was determined by counting the number of intracellular parasites per 10,000 erythrocytes. Identification of haemosporidian parasite species and lineages was limited to molecular methods (but see [49]). Additionally, to parasitic cells, the different leucocytes were counted (leucocyte profile) to determine the H/L ratio (heterophil-to-lymphocyte ratio). Heterophil and lymphocyte cells are the only leucocytes used for further analysis, as these presented the most abundant leucocytes. For every blood smear, all blood cells of a part of the object slide in which blood cells were present as a monolayer were counted until 100 leucocytes were recorded (cf. [50]). This corresponded to an average of 85 ± 39 counted microscopic fields and 17,505 ± 7157 erythrocytes per blood smear.

## 3. Results

Our analysis of the 67 blood samples from columbid birds for the presence of haemosporidian parasites revealed a varying prevalence depending on the method applied. Moreover, prevalence and infection characteristics, such as lineage diversity, varied between host species.

### 3.1. Haemosporidian Parasite Prevalence

According to the results of the nested PCR assay and subsequent sequencing, from all 67 tested samples, 46% tested positive for at least one of the three parasite genera. No infection could be detected in any Stock Dove samples, but 53% of Turtle Dove and 86% of Woodpigeon samples were positive (Table 1). Both birds that had hatched in the calendar year of capture (one Stock Dove and one Turtle Dove) were negative. Comparing the species pairwise due to lower sample size in Woodpigeons and Stock Doves, Stock Doves had a significantly lower overall prevalence than the other species (Fisher’s Exact Test: Stock Dove/Woodpigeon *p* < 0.001; Stock Dove/Turtle Dove *p* < 0.001). There was no significant difference between Turtle Doves and Woodpigeons in overall prevalence (*p* = 0.218).

**Figure 1:**
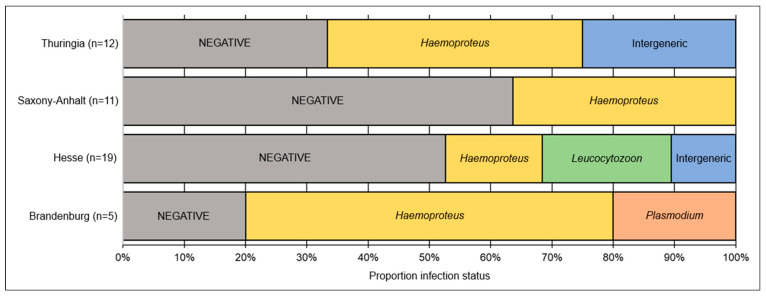
Proportions of negative and positive European Turtle Dove *Streptopelia turtur* blood samples, sampled in four federal states of Germany. Given are the results of nested PCR assay and subsequent Sanger sequencing, split for infections with one haemosporidian genus (*Haemoproteus*, *Plasmodium*, or *Leucocytozoon*) and intergeneric co-infections (either *Haemoproteus* × *Leucocytozoon* (5×) or *Plasmodium* × *Leucocytozoon* (1×)).

In Woodpigeons, only *Haemoproteus* and *Leucocytozoon* infections were detected, with a prevalence of 29% and 71%, respectively. In Turtle Doves, *Haemoproteus* infections were most prevalent (40%), followed by *Leucocytozoon* (19%) and *Plasmodium* (4%). *Haemoproteus* was the only haemosporidian genus found in Turtle Dove samples from all four federal states. *Leucocytozoon* was found only in individuals sampled in Hesse and Thuringia, and *Plasmodium* in Hesse and Brandenburg (Figure 1).

### 3.2. Lineage Diversity Based on Nested PCR Assay

**Figure 2:**
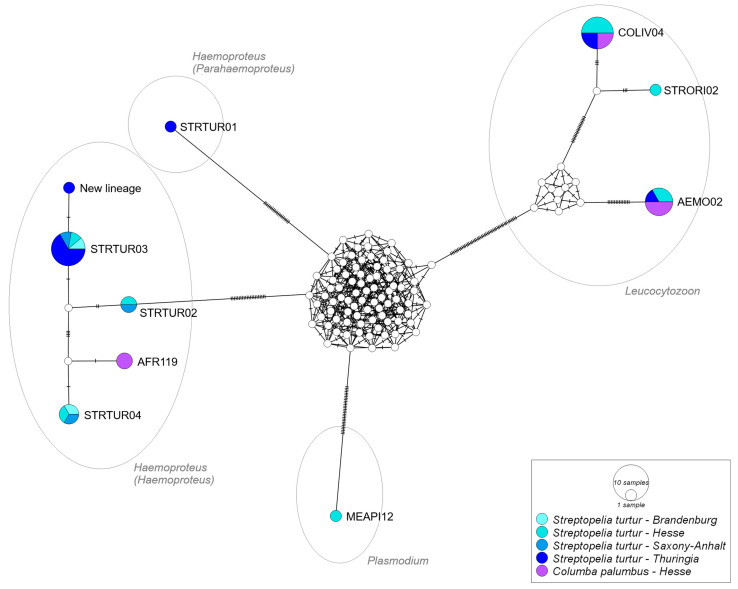
Median-joining network of mitochondrial cytochrome *b* gene lineages (*n* = 34, 478 bp fragment) of haemosporidian parasites *Haemoproteus*, split in subgenera *H*. (*Haemoproteus*) and *H*. (*Parahaemoproteus*), *Leucocytozoon*, and *Plasmodium* infecting European Turtle Doves *Streptopelia turtur* (blue tones) and Common Woodpigeons *Columba palumbus* (purple). Circles represent lineages with circle size proportional to lineage frequencies. Lineage names are noted at the associated circles. One hatch mark represents one mutation.

In total, 10 distinct lineages were determined via the MalAvi comparison. We found three *Leucocytozoon* spp. lineages (L-AEMO02 and L-COLIV04 in Woodpigeons and Turtle Doves; L-STRORI02 in one Turtle Dove) and one *Plasmodium* lineage, namely, P-MEAPI12 in one Turtle Dove sample (Figure 2, GenBank accession number PV402400), which constitutes a previously unknown lineage–host interaction. So far, P-MEAPI12 has only been found in a European Bee-eater *Merops apiaster*, sampled in Portugal (GenBank MT419914, [51]).

The highest lineage diversity, with six lineages, occurred in *Haemoproteus* infections. H-AFR119 was found in Woodpigeons and H-STRTUR01, H-STRTUR02, and H-STRTUR03, as well as H-STRTUR04 in Turtle Doves. One hitherto unknown lineage (GenBank accession number PV402401) was found in a Turtle Dove sample from Thuringia. This new lineage has one mutation difference from H-STRTUR03 and clusters within the *Haemoproteus* (*Haemoproteus*) cluster (Figure 2). All other host–lineage interactions have already been found in previous research (Figure S2).

*Haemoproteus* infections in Turtle Doves have been found in samples from all four federal states (Figure 1 and Figure 2), with H-STRTUR03 occurring in individuals from all federal states. Lineage H-STRTUR04 was present in birds from Hesse, Brandenburg, and Saxony-Anhalt, and H-STRTUR02 in Hesse and Saxony-Anhalt. The *Haemoproteus* (*Parahaemoproteus*) lineage STRTUR01 was found in only one individual from Thuringia, and the *Plasmodium* lineage MEAPI12 in only one Hessian Turtle Dove (Figure 2 and Figure S2). *Leucocytozoon* infections, on the other hand, have been detected only in birds sampled in Hesse and Thuringia. L-AEMO02 and L-COLIV04 have been present in both federal states. The lineage L-STRORI02 was only found in one Turtle Dove sample from Hesse (Figure 2).

In five Turtle Doves and one Woodpigeon (9% of all tested individuals), an intergeneric co-infection with two different parasite genera was present (Figure 1). Most common was a double infection with *Haemoproteus* × *Leucocytozoon*. Present lineage combinations were H-AFR119 × L-AEMO02, H-STRTUR04 × L-AEMO02, H-STRTUR03 × L-AEMO02 and H-STRTUR03 × L-COLIV04. A *Plasmodium* × *Leucocytozoon* infection appeared once (P-MEAPI12 × L-STRORI02). Based on the results of the one-step multiplex PCR, intergeneric infections were a little more prevalent, with 10.4% (seven positive samples). However, in these cases, we do not have any information on lineage identity. An intrageneric co-infection (based on nested PCR) was only found in one Turtle Dove sample from Hesse, namely, with the two *Leucocytozoon* lineages COLIV04 and AEMO02.

### 3.3. Comparison of the Detection Methods

In 82.1% of the cases the results of the one-step multiplex PCR and the nested PCR assay were in total agreement (both negative or same parasite genera), when considering that the one-step multiplex PCR shows the same band height for *Plasmodium* and H. (*Haemoproteus*) infections (cf. [33]). A partial agreement (detection of haemosporidian infection, but differing genus) was present in 7.5% of results. In 10.4% of cases, only one method detected a haemosporidian parasite infection: in two instances, only the one-step multiplex PCR assay, and in five cases, only the nested PCR displayed a band. PCR products of these five cases were Sanger sequenced successfully and could be assigned to lineages unambiguously. The overall prevalence did not differ significantly between both PCR assays (Pearson’s Chi-squared test with Yates’ continuity correction χ^2^ = 0.1, df = 1, *p* = 0.728).

The overall infection prevalence was also not significantly different when comparing the results of blood smear examination and nested PCR assay (χ^2^ = 1.0, df = 1, *p* = 0.325). However, the percentage of agreement of 62.1% (in case of blood smear examination and nested PCR: both negative or both positive for any genus) was lower. Of the 37.9% of the cases of disagreement between the two methods, in 13.6%, only the examination of the corresponding blood smear indicated an infection, while in 24.2% of the cases, only the nested PCR assay indicated an infection.

### 3.4. H/L Ratio and Parasitemia

The mean H/L ratio was 0.71 ± 1.40 with no significant difference between male and female columbid individuals (Wilcoxon rank sum test W = 582.5, *p* = 0.416). However, there was a significant difference between the three species (Kruskal–Wallis χ^2^ = 9.0, df = 2, *p* = 0.011). Turtle Doves had a significantly lower H/L ratio (0.41 ± 0.30) compared to Stock Doves (1.91 ± 3.00; Dunn test Z = 3.0, *p* = 0.004), while both species did not differ from Woodpigeons (0.42 ± 0.15; Z = −0.5, *p* = 0.923 and Z = 1.6, *p* = 0.172, respectively).

**Figure 3:**
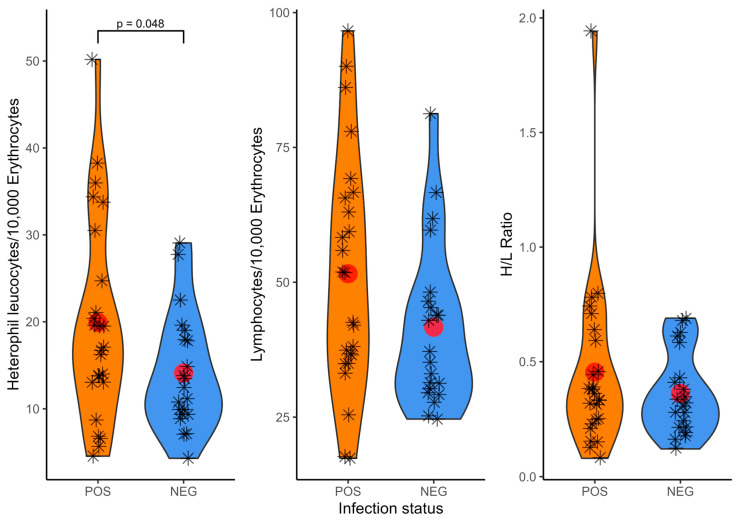
Heterophil and lymphocyte counts and resulting H/L ratio (heterophil-to-lymphocyte ratio) from blood smear counts of European Turtle Doves *Streptopelia turtur* split for infection status with haemosporidian parasites: POS = positive for at least on genus of haemosporidian parasites (*Haemoproteus*, *Plasmodium* and *Leucocytozoon*), NEG = negative, i.e., no infection with one of the haemosporidian genera. Results of infection status based on the results of the nested PCR assay. Red dots indicate median values and a jitter is applied to the displayed data points of count values of individual birds (star symbols).

Due to the smaller sample size for the other two species, further analysis continued with the Turtle Dove samples only. In this species, H/L ratios did not differ between the sexes (Wilcoxon rank sum test W = 309, *p* = 0.246). There was no significant difference in the H/L ratio in infected (0.45 ± 0.37) versus uninfected Turtle Doves (0.36 ± 0.18, W = 228.5, *p* = 0.460, Figure 3). The value for heterophil cells per 10,000 erythrocytes was higher in infected Turtle Doves (19.9 ± 11.5 heterophil/10,000 erythrocytes) as compared to uninfected individuals (14.1 ± 6.7, *t*-test t = −2.038, df = 44, *p* = 0.048, Figure 3). Similarly, the number of lymphocytes per 10,000 erythrocytes was also higher in infected individuals (51.6 ± 21.7 vs. 41.7 ± 15.1); however, this trend was not significant (t = −1.767, df = 44, *p* = 0.084, Figure 3).

In 21 blood smear samples of Turtle Doves with intracellular parasites (Table 1), the parasitemia ranged from 0.6 to 7.9 parasites/10,000 erythrocytes, with a mean of 2.3 ± 1.7 parasites/10,000 erythrocytes. The H/L ratio did not increase significantly with an increasing parasitemia (Spearman’s rank correlation S = 1399.9, rho = 0.091, *p* = 0.695).

## 4. Discussion

### 4.1. Pattern of Infection and Lineage Diversity

In agreement with previous studies [31,33], we found a high overall prevalence in Woodpigeons, followed by Turtle Doves, and an absence of infections in Stock Doves. As already discussed in the earlier studies, this pattern might be partly attributed to the differing nesting behaviors, with cavity-nesting Stock Doves being less exposed to dipteran vectors than open-nesting Turtle Doves and Woodpigeons. A similar pattern regarding infection propensity and nest type was found in different avian communities at other locations. Rodriguez et al. [52] examined 24 bird species in the Colorado Rocky Mountains and found that open-cup nesting species had higher *Haemoproteus* prevalence than cavity or ground nesters. A study from the Brazilian Pantanal, including 149 avian species, demonstrated that birds that build open-cup nests were more likely to be infected by *Haemoproteus* parasites compared to birds with closed-cup nests, nests in tree holes, or birds utilizing cavities [53].

When considering the prevalence of the individual haemosporidian parasite genera, the results of this study are also in line with those of [33]. While *Leucocytozoon* infections were most prevalent in Woodpigeons, *Haemoproteus* infections dominated in Turtle Doves, and *Plasmodium* occurred only in Turtle Doves, with a low prevalence. Thus, we recorded a higher infection prevalence with *Leucocytozoon* and lower infection prevalence of Haemoproteidae (*Plasmodium* and *Haemoproteus*) in residents or short-distance migrants (Woodpigeons) compared to higher haemoproteid parasite prevalence in long-distance migrants (Turtle Doves). These results are in accordance with previous findings, as *Leucocytozoon* is transmitted to birds predominantly at the breeding grounds, whereas for long-distance migrants breeding in the Northern hemisphere, haemoproteid parasite transmission likely takes place mainly during the nonbreeding period [26,37,54,55,56]. However, defining the actual transmission location in avian haemosporidian parasites is challenging [57,58]. To designate actual transmission areas, it would be necessary to sample juvenile birds that have not yet performed any migratory movements (cf. [57,59]).

Pathogen exposure may be an important driver of site selection during the nonbreeding period, as some migration strategies are thought to be the result of actively avoiding parasite-rich habitats by choosing a nonbreeding site with lower prevalence of parasites [60,61,62,63,64,65]. In migratory hosts with rather large distribution ranges, the local breeding population can have distinct nonbreeding areas with no or little overlap between populations (i.e., high degree of migratory connectivity). In such cases, there might be host–parasite interactions specific to populations, which, consequently, may result in population-specific parasite prevalence and diversity [66]. Therefore, haemosporidian parasites could serve as possible markers of geographic origin to track migratory patterns [67].

For Turtle Doves following the Western Afro-Palearctic flyway (e.g., individuals from the UK and France), strong migratory connectivity is suggested [39]. However, in line with population genetic analyses showing no clear genetic structure, for those following the Eastern flyway (e.g., individuals from the Czech Republic and Greece), a weak connectivity due to flyway permeability is assumed [39,68,69]. Zwarts et al. [70] investigated migratory connectivity based on recoveries and recaptures in Africa between 4° and 35° N, revealing hardly any overlap in the west–east distribution in Africa of Turtle Doves breeding in different longitudinal zones in Europe. This indicates a strong migratory connectivity to broad regions, also suggesting winter regions in the Western, Central, and Eastern sub-Sahara mirroring the different flyways [70]. Regarding migration flyways, a migratory divide between the Czech Republic and Germany was proposed for Turtle Doves [38,39].

Within this study, we checked for possible population-specific haemosporidian patterns on a smaller scale within Germany, spanning an east–west gradient (from the federal states Hesse to Brandenburg) through which the suggested migratory divide should pass [39]. The prevalence of *Plasmodium* was generally too low for any more specific interpretations of patterns. *Leucocytozoon* infections have been found in Turtle Doves from Hesse and Thuringia, representing the two westernmost federal states included. Except for one Hessian individual infected with the lineage L-STRORI02, the lineages L-COLIV04 and L-AEMO02 occurred in individuals from both federal states. As these lineages were also present in resident or short-distance migrating Woodpigeons, they are likely transmitted on the breeding grounds; thus, breeding ground characteristics are more likely to shape this pattern rather than characteristics at the nonbreeding sites. As such, *Leucocytozoon*-transmitting blackflies (family Simuliidae) need running water to complete their life cycle, while dipterans transmitting *Haemoproteus* and *Plasmodium* are associated with standing water bodies [71,72]. In this context, it would be interesting to sample Woodpigeons from Saxony-Anhalt and Brandenburg to check whether L-COLIV04 and L-AEMO02 are also absent there. However, a sufficiently large sample size would be required to answer this question.

Only infections with *Haemoproteus* spp. were found in birds from all four federal states. Within the five *Haemoproteus* lineages found in Turtle Doves, there is no clear pattern with regard to the geographical distribution of lineages specific to breeding and wintering sites or flyways. In other migrants, such as Common Yellowthroats *Geothlypis trichas*, haemosporidian parasite analyses suggest some geographic structuring of *Plasmodium* lineages [67]. However, in this case, the authors state their limited utility for determining migratory connectivity. Bensch and Åkesson [73] found no clear geographic structuring of *Haemoproteus* lineages in Willow Warbler *Phylloscopus trochilus*. Examining the haemosporidian fauna of Collared Sand Martins *Riparia riparia* combined with geolocator tracking, Hahn et al. [66] showed a differential annual prevalence in local breeding populations, indicating population-specific host–parasite interactions. However, considerable between-year variation in prevalence and the detection of numerous new lineages hampered conclusions as to whether population-specific migration patterns resulting in different nonbreeding regions cause differential blood parasitism in Sand Martins. Generally, it is known that the prevalence and diversity of haemosporidian infections can vary with age and sex, and that annual and seasonal fluctuations occur (e.g., [74,75]), which should be taken into account when interpreting results.

### 4.2. Differences in Haemosporidian Parasite Detection Methods

Different methodological approaches for parasite detection led to differing prevalence, even if the difference was not significant when comparing the methods pairwise. The lowest prevalence was detected by blood smear counts (36.4%), and the highest derived from the nested PCR assay (46.3%). The same pattern, i.e., finding lower prevalence via blood smear examination than by PCR assays, was shown in other studies (e.g., [76,77,78,79]. For instance, in a study on passerine birds, 19 of 77 *Haemoproteus* infections (25%) and 31 of 57 *Plasmodium* infections (54%) detected by the PCR method were not detected when examining ca. 10,000 red blood cells from smears microscopically [76]. The absence of gametocytes in blood smears of individuals tested positive by PCR could be due to light gametocyte parasitemia, or it might be explained by DNA amplification of circulating sporozoites or the presence of parasite remnants that aborted development within the host [80,81,82]. Nevertheless, blood smears provide critical information for distinguishing between abortive and successful chronic infections [83] and on the intensity of infection [76,82]. It provides the information that our sample set most likely contained only chronic infections with low parasitemia [33]. Furthermore, the risk of having false positives is known to be much lower in microscopic examination of blood smears as compared to detection via PCR [84]. However, we carried out Sanger sequencing of the positive results of the nested PCR, which would most likely have detected any false positives.

In wildlife, co-infections of different species and lineages of haemosporidian parasites predominate and are often associated with high virulence [85]. Applying more than one PCR protocol, in particular, the multiplex PCR technique, permits simultaneous amplification of more than one target of interest in a single PCR reaction by incorporating several primer pairs, which can be helpful for the detection of co-infections [43,78,79]. In our dataset, the one-step multiplex PCR had a slightly higher prevalence for intergeneric co-infections as compared to the nested PCR assay. However, we do not have more specific information on such intergeneric co-infections, as the PCR products of the one-step multiplex PCR cannot be used for lineage determination with the MalAvi database. The same applies to intrageneric co-infections. Overall, it is likely that the prevalence of co-infections in our study is still underestimated (cf. [85]).

### 4.3. H/L Ratio and Infection Status

Analysis of blood is an important component in the assessment of the health status of birds [86,87]. Lymphocytes and heterophil leucocytes are the two most abundant leucocytes in birds, playing an essential important role in cell-mediated as well as humoral adaptive and innate immune defense, respectively. The ratio of heterophil to lymphocyte cells (H/L ratio) reflects the state of the immune system [88,89]. As elevated H/L ratio constitutes a corticosterone-mediated response to external stressors, it is generally acknowledged as a proxy for the intraspecific variation in physiological stress [20,88]. The H/L ratio is known to change in response to external stressors, e.g., inclement climatic, pollution, increased breeding effort, or parasitic infestation [20,21,90,91]. Baseline data for the species are critical to recognize and interpret ratio changes. Thus, without published reference values for the species, it can be difficult to interpret H/L ratios [92]. In a study including seven species of Columbiformes, a wide range of H/L ratio values (0.19 to 64.67) was observed [86]. Our mean value of 0.71 ± 1.40 is within an observed range of H/L ratios in other columbid species, e.g., Diamond Doves *Geopelia cuneata* (0.54 ± 0.21, [93]), Eurasian Collared Dove *Streptopelia decaocto* (0.76 ± 0.02, [87]; 0.63 ± 0.14, [94]), and African Collared Dove *Streptopelia roseogrisea* (1.05 ± 0.04, [87]). However, profiles can vary not only interspecifically but also intraspecifically with sex, season, age, and habitat [25,92]. Therefore, in our dataset, the H/L ratio in infected compared to uninfected conspecifics might have been influenced by various individual factors and external stressors, such as other diseases apart from haemosporidian infections. To check if haemosporidian infection alone alters leucocyte profile, targeted infection experiments would be necessary, similar to an experimental infestation with *Isospora* coccidians performed in House Sparrows [95].

In general, parasites are assumed to drain resources of hosts, and the effects of parasite load on H/L ratios have long been investigated in birds. While some study results imply that parasites elevate H/L ratios (e.g., [96,97,98], others report no evidence of such. Concurrent to our results, Dimitrov et al. [99] found no significantly higher H/L ratio in Rosy Starlings *Pastor roseus* infected with haemosporidian parasites, and Santiago-Alarcon et al. [25] found no association between haemosporidian infections and H/L ratio in House Sparrows *Passer domesticus*. While the H/L ratio did not significantly predict haemosporidian infection status in three species of neotropical migrants [24], heterophil and lymphocyte counts were higher in infected than in uninfected Gray Catbirds *Dumetella carolinensis*, similar to the higher heterophil per 10,000 erythrocytes counts in our infected Turtle Doves. Yellowhammers *Emberiza citrinella* infected with *Haemoproteus* had an elevated standardized white blood cell count, but a reduced H/L ratio [22]. A study on nestlings of the same three Columbid species as analyzed here found no evidence for an association between infection status and immune status based on white blood cell counts [31]. These results indicate that some birds with chronic blood parasite infections can have higher leucocyte profiles than uninfected conspecifics. One interpretation of this pattern is that chronic or latent infections might elicit long-term activation of innate immune defense (cf. [22,24]). Chronic infections, defined as recovered infections with low parasitemia as observed in our dataset, are common in wild birds and may be persistent throughout the host’s entire life [26]. Certain avian host species might be more tolerant of chronic infections due to differences in life history and behavioral traits. These differences could affect the signal of elevated immune defense following an initial infection [24], which might explain the observed differences in the change in leukocyte profiles, including H/L ratio values, in different host species.

## 5. Conclusions

In conclusion, the present study contributes to our understanding of the haemosporidian parasite diversity circulating in birds of non-passeriform orders, confirming previous results of infection patterns and revealing new host–lineage interactions in free-ranging species of Columbiformes. However, lineage occurrence in Turtle Dove samples did not reflect population-specific nonbreeding areas; thus, it does not seem suitable as a marker of geographic origin to track migratory patterns. Future work is warranted to shed more light on possible hitherto unknown population-specific parasite prevalence and diversity and dynamics of haemosporidian parasitism in Columbiformes.

## Figures and Tables

**Table 1:** Number of blood samples collected in Germany in 2023 and 2024, split by species and federal state, and respective overall infection prevalence (the three evaluated haemosporidian genera combined) for each analysis method.

Host Species	Sampling Site ^a^	Sample Size FTA	Prevalence Nested PCR	Prevalence One-Step Multiplex	Sample Size Blood Smear	Prevalence Blood Smear Examination
Stock Dove *C. oenas*		13	0%	0%	13	0%
	Hesse	4	0%	0%	4	0%
	Thuringia	9	0%	0%	9	0%
Woodpigeon *C. palumbus*	Hesse	7	85.7%	85.7%	7	42.9%
Turtle Dove *S. turtur*		47	53.2%	46.8%	46	45.7%
	Brandenburg	5	80.0%	80.0%	5	0%
	Hesse	19	47.4%	36.8%	18	44.4%
	Saxony-Anhalt	11	36.4%	27.3%	11	36.4%
	Thuringia	12	66.7%	66.7%	12	75.0%
**All samples**		**67**	**46.3%**	**41.8%**	**66**	**36.4%**

^a^ See Figure S1 for the position of sampling sites within Germany.

## Data Availability

Sequences are deposited in GenBank under accession numbers PV402400 and PV402401. Raw data are available by request from the corresponding author.

## Supplementary Materials

The following supporting information can be downloaded at: https://www.mdpi.com/article/10.3390/microorganisms13061305/s1, Table S1 Sequences of primer pairs used for detection of haemosporidian parasites in DNA isolated from host blood. Two PCR assays have been applied: Nested PCR with an initial and two specific PCR reactions (Hellgren et al. [42]) and one-step multiplex PCR (Ciloglu et al. [43]). Figure S1 Catching sites of sampled columbids within four federal states, namely Hesse, Thuringia, Saxony-Anhalt and Brandenburg, within Germany, split for both sampling years 2023 (blue) and 2024 (red). Figure S2 Median-joining network of mitochondrial cytochrome *b* gene lineages (*n* = 163, 478 bp fragment) of haemosporidian parasites *Haemoproteus*, split in subgenera *H*. (*Haemoproteus)* and *H.* (*Parahaemoproteus*), *Leucocytozoon* and *Plasmodium*. Host species and samples origin (this study: ‘new’, darker tones or previous study: lighter tones, Schumm et al. [33]) are colour coded. Circles represent lineages with circle size proportional to lineage frequencies. Lineage names are noted at the associated circles. One hatch mark represents one mutation.
