# Supplementary material for: Avian Haemosporidian Parasites in Three Wild Columbids from Germany
Source: Microorganisms. 2025 Jun 4;13(6):1305. doi: 10.3390/microorganisms13061305 (PMC12195335; doi:10.3390/microorganisms13061305)
Supplement: Supplementary file 1 [file microorganisms-13-01305-s001.zip › microorganisms-3622017-supplementary.pdf]

- Supplementary material –

Avian haemosporidian parasites in three wild columbids from Germany

Yvonne R. Schumm[1], Celine Frank[1], Uta Gerz[1], Hannes Ruß[1], Benjamin Metzger[2] & Petra Quillfeldt[1]*

*Corresponding author: Petra.Quillfeldt@bio.uni-giessen.de

Table S1 Sequences of primer pairs used for detection of haemosporidian parasites in DNA isolated from host blood. Two PCR assays have been applied: Nested PCR with an initial and two specific PCR reactions (Hellgren et al. 2004) and one-step multiplex PCR (Ciloglu et al. 2019).

Primer pair	Target gene	Primer sequence (5' to 3')	Annealing Temperature [°C]	PCR assay
HaemNFI<br>HaemNR3	Cyt *b*	CATATATTAAGAGAAITATGGAG<br>ATAGAAAGATAAGAAATACCATTC	50	Initial Nested
HaemF<br>HaemNR3	Cyt *b*	ATGGTGCTTTCGATATATGCATG<br>GCATTATCTGGATGTGATAATGGT	50	Specific Nested
HaemFL<br>HaemR2L	Cyt *b*	ATGGTGTTTTAGATACTTACATT<br>CATTATCTGGATGAGATAATGGIGC	50	Specific Nested
PMF<br>PMR	Non-coding region mtDNA	CCTCACGAGTCGATCAGG<br>GGAAACCGGCGCTAC	59	One-Step Multiplex
HMF<br>HMR	Cyt *b* + non-coding region mtDNA	ATTGGATGTCAATTACCACAATC<br>GGGAAGTTTATCCAGGAAGTT	59	One-Step Multiplex
LMF<br>LMR	COX1	TGGAACAATAATTGSATTATTTACAYT<br>AACATATCATATTCCATCCATTTAGATTA	59	One-Step Multiplex

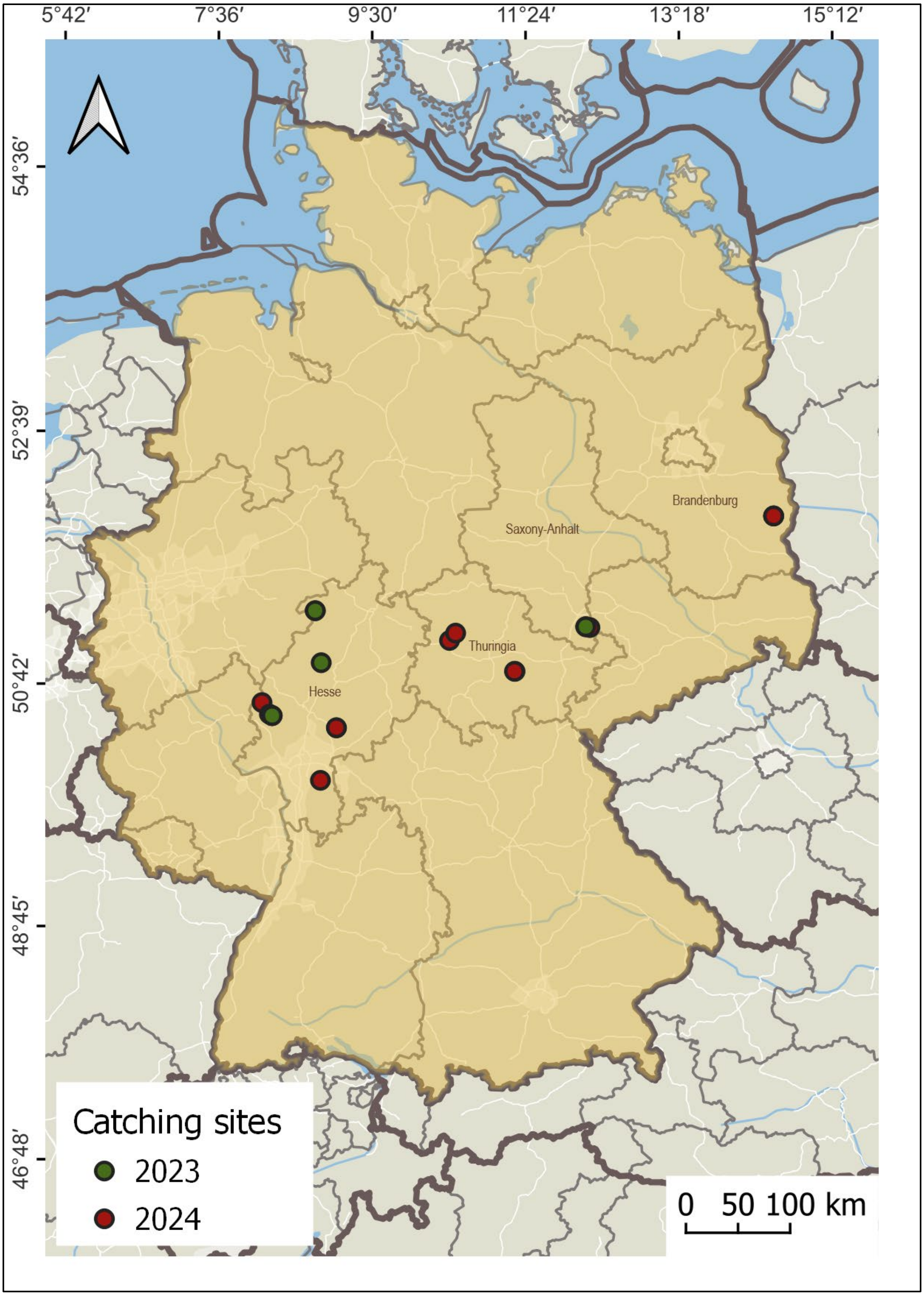

Figure S1 Catching sites of sampled columbids within four federal states, namely Hesse, Thuringia, Saxony-Anhalt and Brandenburg, within Germany, split for both sampling years 2023 (green circles) and 2024 (red circles).

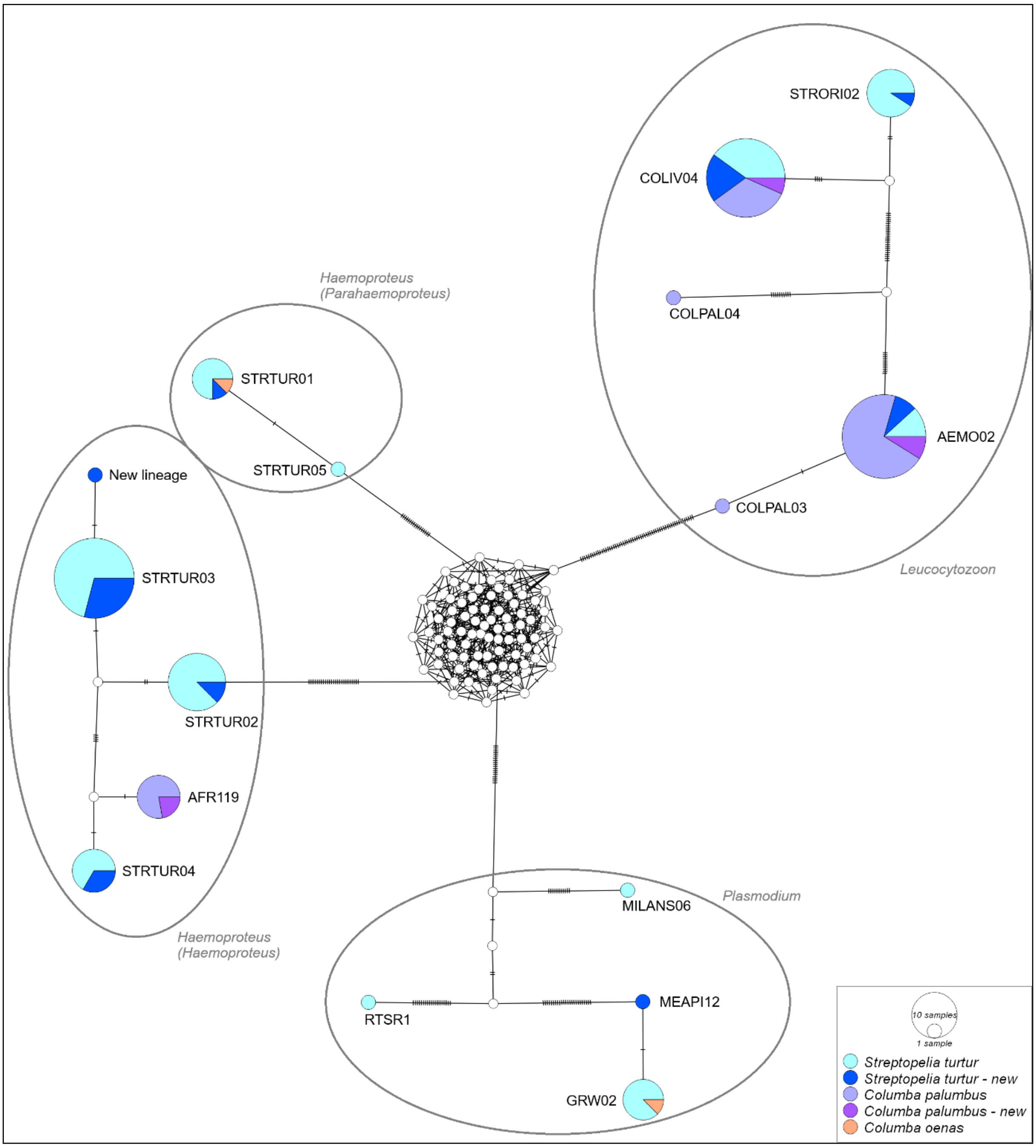

Figure S2 Median-joining network of mitochondrial cytochrome *b* gene lineages (n = 163, 478 bp fragment) of haemosporidian parasites *Haemoproteus*, split in subgenera *H.* (*Haemoproteus)* and *H.* (*Parahaemoproteus*), *Leucocytozoon* and *Plasmodium*. Host species and samples origin (this study: 'new', darker tones or previous study: lighter tones, Schumm et al. 2021) are colour coded. Circles represent lineages with circle size proportional to lineage frequencies. Lineage names are noted at the associated circles. One hatch mark represents one mutation.
